# Construction and Validation of a Novel Immune Checkpoint-Related Model in Clear Cell Renal Cell Carcinoma

**DOI:** 10.1155/2022/9010514

**Published:** 2022-12-30

**Authors:** ZhiXiang Fan, XinXin Sun, Kun Li, YanYan Zhang, ShiKai Zuo, CanCan Li, Shi Wan, DongMei Huang

**Affiliations:** ^1^The Department of Obstetrics and Gynecology, The Second Affiliated Hospital of Zhengzhou University, Henan, Zhengzhou 450014, China; ^2^The Department of Oncology, The Second Affiliated Hospital of Zhengzhou University, Henan, Zhengzhou 450014, China

## Abstract

**Background:**

With the highest mortality and metastasis rate, kidney renal clear cell carcinoma (KIRC) is one of the most common urological malignant tumors and not sensitive to chemotherapy and radiotherapy. Immunotherapy, which proves to be effective and a big progression, such as PD-1/PD-L1 inhibitors, is not sensitive to all KIRC patients. To predict prognosis and immunotherapy response, a novel immune checkpoint gene- (ICG-) related model is essential in clinics.

**Methods:**

From the public database-downloaded dataset, a novel ICG-related model for predicting prognosis and immunotherapy response in KIRC patients was built up and verified with R packages and Cox regression analysis. The Kaplan-Meier curve was plotted.

**Results:**

39 ICGs were identified to have different expression in KIRC patients and enriched in immune-related biological pathways and activities. Three ICGs (CTLA4, TNFSF14, and HHLA2) were screened to generate KIRC-ICG model. The KIRC-ICG model was verified to be effective. With conducting KIRC-SYS model, KIRC-ICGscore was verified to be an independent factor regardless of age, gender, stage, grade, and TNM stage. Compared to the ICG-low subgroup, the ICG-high subgroup had more immune activities. KIRC-ICGscore was significantly positively correlated with the expression of Treg markers. KIRC-ICG model could also be reliable to predict immunotherapy response.

**Conclusion:**

The KIRC-ICG model was reliable to predict prognosis and immunotherapy response for KIRC patients and could be an independent factor regardless of clinical characteristics.

## 1. Introduction

Taking up 80% of renal carcinoma, kidney renal clear cell carcinoma (KIRC) is one of the most common urological malignancies [1]. Patients with renal clear cell carcinoma have the highest mortality and metastasis rate [2–4] and are not sensitive to chemotherapy and radiotherapy [5]. Combined with traditional cancer treatment, tumor immunotherapy has high efficacy and safety, which can significantly improve the survival rate of patients [6].

Despite significant progression in immunotherapy [7], there are still a proportion of KIRC patients who are not sensitive to these inhibitors due to immunosuppression [8]. Tumor immunosuppression refers to the inhibition of the host immune response to tumor antigens under the action of some factors, resulting in tumor cells escaping from the surveillance of the body's immune system, rapidly dividing and proliferating in the body, and accelerating the deterioration of tumors [9, 10]. These factors include a series of molecules expressed on immune cells that regulate the extent of immune activation, called immune checkpoints. For example, PD-1, a type I transmembrane glycoprotein expressed mainly on the surface of T cells, inhibits the activation and proliferation of T cells by binding to PD-L1 [11]. Nevertheless, due to the diversity of immune checkpoints, different types of cancer can express different immune checkpoints. To predict prognosis and immunotherapy response, a new immune checkpoint gene- (ICG-) related model is essential in clinics for KIRC patients.

To address this issue, we identified ICG-related differential genes (DEGs) and constructed a new KIRC-ICG model. Meanwhile, we also verified the KIRC-ICG model performance in prognosis and immunotherapy response effectiveness. It would provide a new vision for improving accuracy in immunotherapy in KIRC patients.

## 2. Material and Methods

### 2.1. Data Collection

The KIRC data (RNA-seq data and clinical data) from the TCGA database were downloaded from the University of California Santa Cruz's official webpage (UCSC database) (https://xenabrowser.net/datapages/). TCGA-KIRC dataset included RNA transcript of 531 KIRC patients and 72 paracarcinoma tissues, in which we obtained 515 KIRC patients with survival time greater than 30 days. To supplement the calculations, we also obtained the RNA-seq data from normal people in The Genotype-Tissue Expression (GTEx) databases. In addition, we got the RECA-EU dataset with expression profiling of KIRC patients as the validation cohort from the International Cancer Genome Consortium (ICGC) database. The complete dataset of 56 renal cell carcinoma patients with immune therapy information was downloaded from the IMvigor210 (http://research-pub.gene.com/IMvigor210CoreBiologies) [12]. 47 immune checkpoint genes (ICGs) were collected from published literature (Table 1) [13].

### 2.2. Identification of ICGs

The “limma” package was applied to filter immune checkpoint genes in both TCGA-KIRC and RECA-EU datasets by setting *p* < 0.05 and |log2FoldChange| > 1 as the threshold. The GeneMANIA was used to explore the protein network in these ICGs from multiple perspectives [14].

### 2.3. Construction of Prognostic Risk Model

Univariate Cox regression analysis was used to identify ICGs which were related to overall survival (OS). Next, through a multivariate Cox analysis (stepwise model), we further filtered these ICGs affecting prognosis and built up the prognostic risk model with the lowest Akaike information criterion (AIC) value. AIC is a standard for assessing the complexity of statistical models and measuring the goodness of fit. Based on the coefficient and expression value of ICGs, the risk score of each sample was estimated. The equation was as follows:

(1) $$\begin{array}{c} \text{KIRC}-\text{ICGscore}=\overset{n}{\underset{i=1}{\sum }}\exp i*\text{coef}i. \end{array}$$

To evaluate the model, the “survivalROC” package was used to plot time-dependent ROC curves. Based on the KIRC-ICGscore, we classified KIRC patients (*n* = 515) into the ICG-high (*n* = 258) and ICG-low subgroups (*n* = 257).

## 3. Analysis of Tumor Immune Microenvironment (TIME) in KIRC-ICG Subgroups

The “GSVA” and “Estimate” packages were used to analyze the differences in the TIME status between the two KIRC-ICG subgroups [15, 16]. The Tumor Immune Dysfunction and Exclusion (TIDE) database (http://tide.dfci.harvard.edu/) was applied to predict the effect of immunosuppressive therapy on the two KIRC-ICG subgroups [17].

## 4. Immune Clustering Based on ssGSEA

The ssGSEA algorithm was based on the 29 immune-associated gene sets which represented diverse immune cell types, functions, and pathways [18]. We applied the ssGSEA algorithm via R packages “GSVA” to comprehensively assess the immunologic characteristics of every sample included in the TCGA-KIRC set and performed an unsupervised hierarchical clustering algorithm to divide these samples into Immune_H and Immune_L clusters based on the ssGSEA results.

## 5. GSVA for Functional Annotation

Gene Set Variation Analysis (GSVA) enrichment was applied to explore the pathway differences between two clusters by the R packages “GSVA” and “Limma” [19].

### 5.1. Statistical Analysis

The Wilcoxon test was performed to screen ICGs. The log-rank test was used in the Kaplan-Meier curve. The GSEA was performed to explore potential biological pathways by setting normal *p* value <0.05 and FDR (false discovery rate) *q* value <0.25 as the threshold. All statistical analyses were conducted using the R (4.0.3) program, and *p* < 0.05 was considered significant.

## 6. Results

### 6.1. Identification of ICGs in KIRC Patients

**Figure 1:**
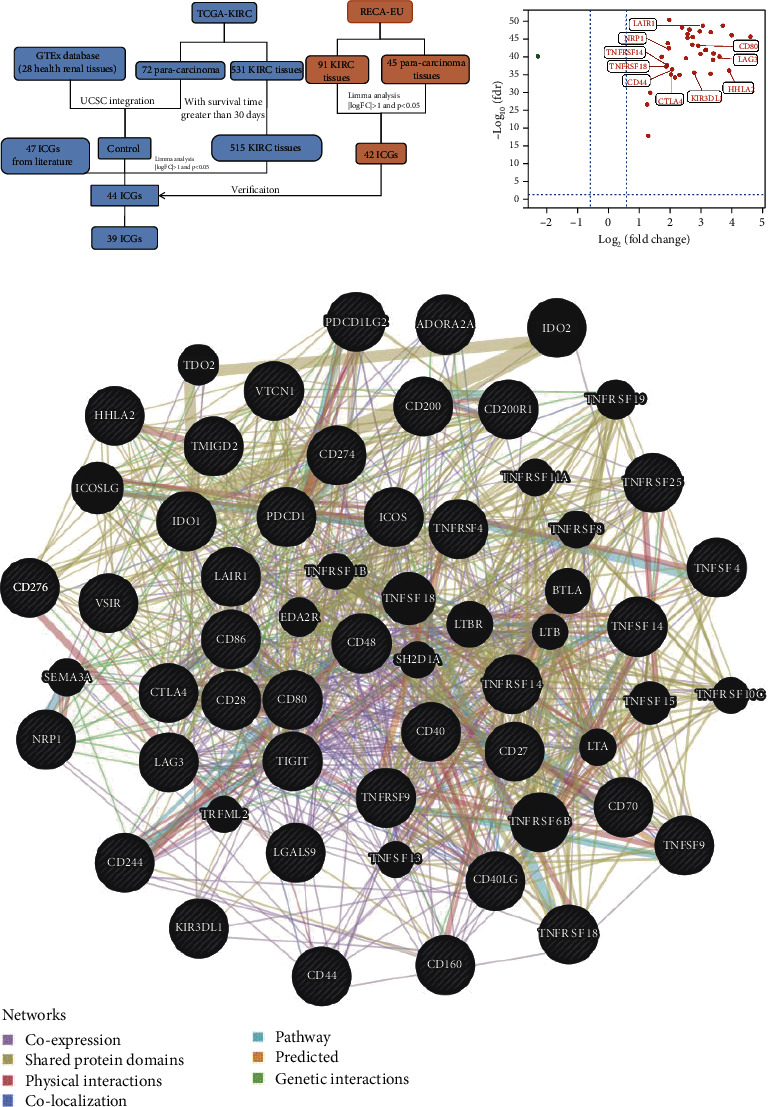
Identification of the ICG-related DEGs in KIRC patients. (a) Data flowchart to obtain 39 ICGs from TCGA-KIRC and RECA-EU datasets. (b) Volcano plot of 39 ICG-related DEGs. The red dot represents upregulation genes, and the green dot represents downregulation genes. (c) The genes interaction network of 39 ICGs. ICGs: immune checkpoint genes; DEGs: differential genes.

Within 47 ICGs collected from published literature, 44 ICGs were discovered differentially expressed by comparing 515 KIRC tissues with 72 paracarcinoma tissues and 28 healthy renal tissues, in which 39 ICGs were validated in RECA-EU cohort (Figure 1(a)). Inside these 39 ICGs, there are 38 upregulated genes and 1 downregulated gene (Figure 1(b)). Subsequently, to understand the correlation between 39 ICGs, we constructed a gene interaction network by GENEMANIA. The interaction network of these ICGs revealed physical contacts on “co-localization,” “shared protein domains,” “co-expression,” and “potential functional interactions on pathways” (Figure 1(c)). It further confirmed that these ICGs may not work alone but interact in close association.

### 6.2. Construction of the ICG-Related Prognostic Risk Model in KIRC

**Figure 2:**
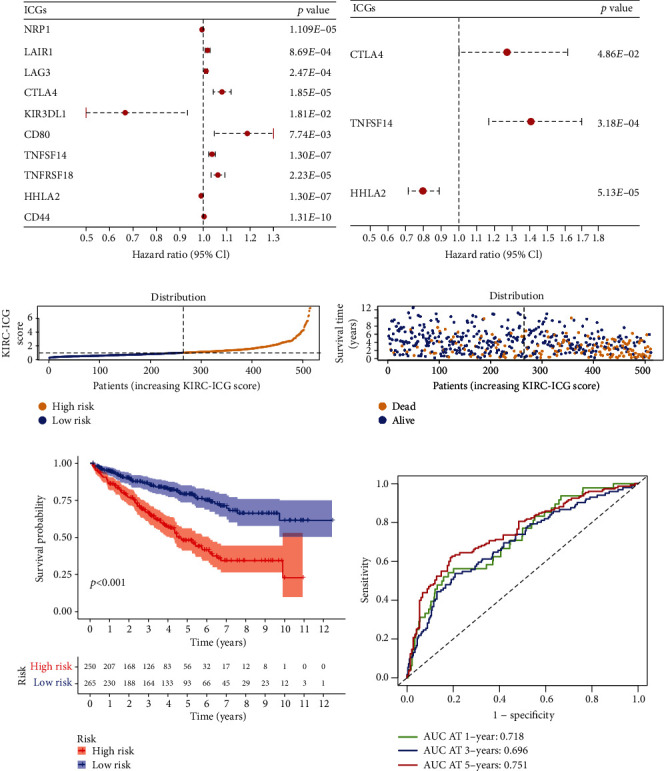
Construction and validation of the ICG-related prognostic risk model in KIRC. (a) The univariate Cox analysis. (b) The multivariate Cox analysis. (c) Distribution of KIRC-ICGscore. (d) Distribution of survival status. (e) The Kaplan-Meier analysis on the two KIRC-ICG subgroups. (f) The ROC curve of the KIRC-ICG model at 1, 3, and 5 years. ICGs: immune checkpoint genes.

By performing the univariate Cox analysis, we screened out 10 ICGs significantly associated with OS (*p* < 0.05) (Figure 2(a)) and subjected them to the multivariate Cox analysis. After the multivariate Cox analysis (stepwise models), there were finally three genes (CTLA4, TNFSF14, and HHLA2) included in the prognostic risk model (KIRC-ICG model) with the lowest AIC value (775.17) (Figure 2(b)). The equation was as follows:

(2) $$\begin{array}{c} \text{KIRC}-\text{ICGscore}=\left(0.240\times \text{CTLA}4\right)+\left(0.341\times \text{TNFSF}14\right)-\left(0.227\times \text{HHLA}2\right). \end{array}$$

**Figure 3:**
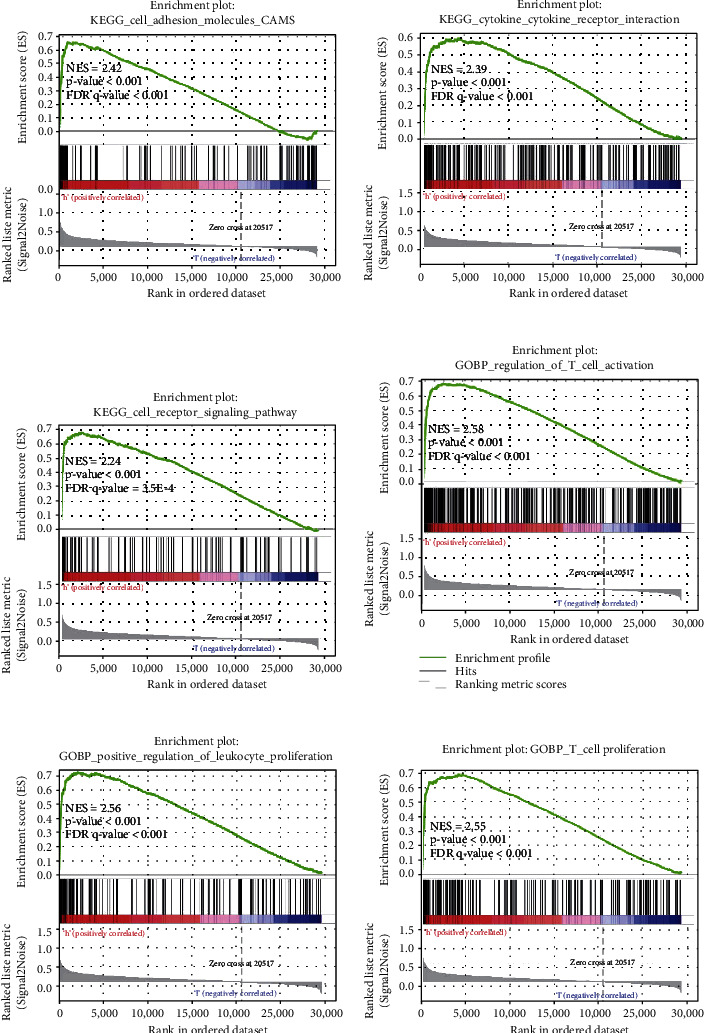
GSEA enrichment analysis. The ICG-high subgroup related to KEGG (a–c) and GO (d–f) pathway enrichment. ICGs: immune checkpoint genes.

With the median value of the KIRC-ICGscore, we classified KIRC patients (*n* = 515) into the ICG-high (*n* = 258) and ICG-low subgroups (*n* = 257). The distribution of the KIRC-ICGscore and survival status between the two KIRC-ICG subgroups were shown in Figure 2(c). There were more death samples with higher KIRC-ICGscore (Figure 2(d)). The Kaplan-Meier analysis confirmed a lower OS rate in the ICG-high subgroup (*p* < 0.05, Figure 2(e)). The AUCs of time-dependent ROC curve at 1, 3, and 5 years were 0.718, 0.696, and 0.751, which indicated that the KIRC-ICG model was reliable for predicting the prognosis of KIRC patients (Figure 2(f)). To illustrate the underlying mechanism of the survival difference between the ICG-high and ICG-low subgroups, GSEA was applied. The results related to KEGG and GO pathways showed that the ICG-high subgroup had more immune activities (Figures 3(a)–3(f)).

### 6.3. Clinical Application of the KIRC-ICG Model

**Figure 4:**
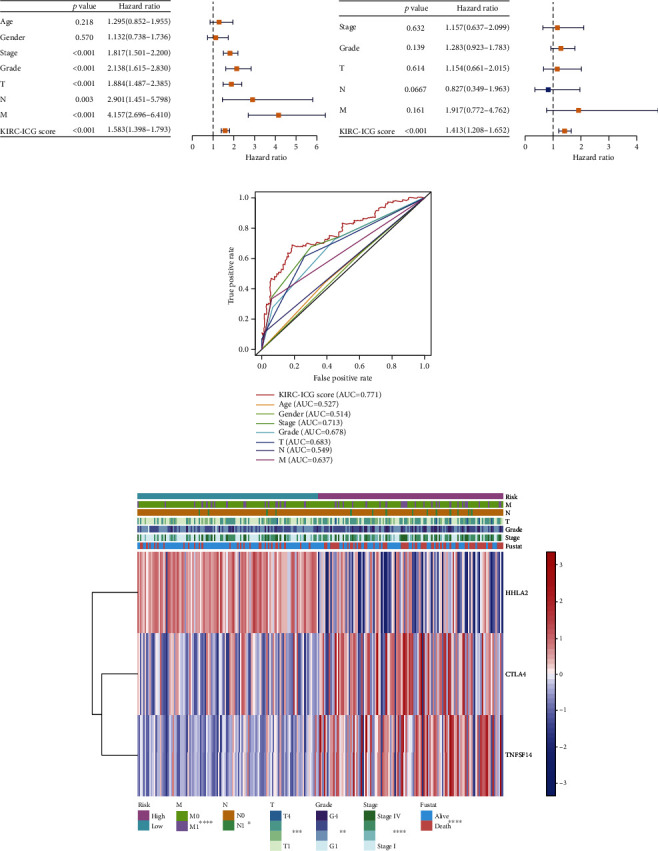
Construction of the systematic prognostic risk model in KIRC. (a) Univariate Cox analysis. (b) Multivariate Cox analysis. (c) The ROC curves of different clinical characteristics. (d) Heat map of distribution of different clinical characteristics including KIRC-ICGsore. ^∗^*p* < 0.05, ^∗∗^*p* < 0.01, ^∗∗∗^*p* < 0.01, and ^∗∗∗∗^*p* < 0.0001.

**Figure 5:**
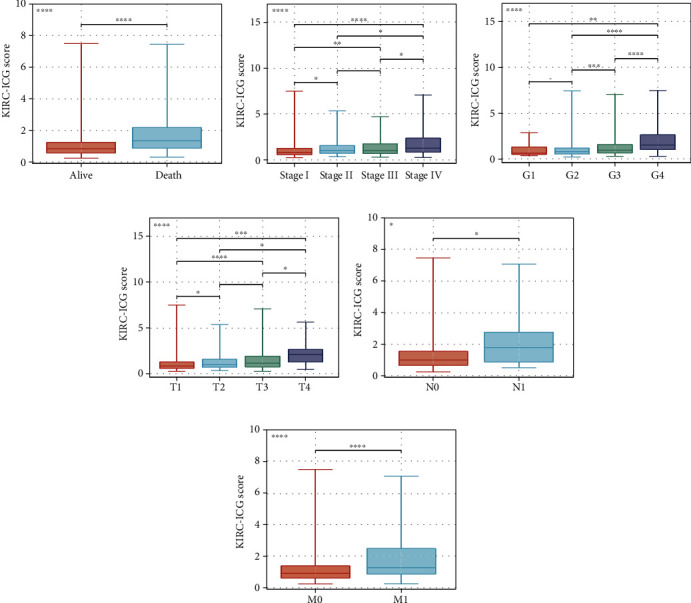
Statistical discrepancy based on KIRC-ICGscore in subsets of different clinical characteristics; (a) survival status; (b) stage, (c) grade; (d) T stage; (e) N stage; (f) M stage. ^∗^*p* < 0.05, ^∗∗^*p* < 0.01, ^∗∗∗^*p* < 0.001, and ^∗∗∗∗∗^*p* < 0.0001.

To evaluate the KIRC-ICGscore in the clinic, six characteristics (age, gender, stage, grade, TNM stage, and KIRC-ICGscore) were enclosed to build up the systematic prognostic risk model named KIRC-SYS model by conducting univariate Cox regression and multivariate Cox regression analyses. As shown in Figures 4(a)–4(b), the KIRC-ICGscore was identified as an independent risk factor for OS. And the AUC value for the KIRC-ICGscore was 0.771 (Figure 4(c)), which was superior to other clinicopathological parameters, especially the TNM stage. All these features indicated that the KIRC-ICGscore was systematically dependable in predicting the prognosis of KIRC patients. Moreover, we drew a distribution heat map of clinicopathological characteristics and three ICGs between the two KIRC-ICG subgroups (Figure 4(d)) and discovered that TNM stage, stage, grade, and survival status (fustat) were different between the two KIRC-ICG subgroups (all *p* < 0.05). With the KIRC-ICGscore as a continuous variable, we further demonstrated that the high KIRC-ICGscores were linked to poorly differentiated tumors, advanced TNM stages, and death (Figure 5). It further revealed that the high KIRC-ICGscores might be associated with the progression of KIRC.

**Figure 6:**
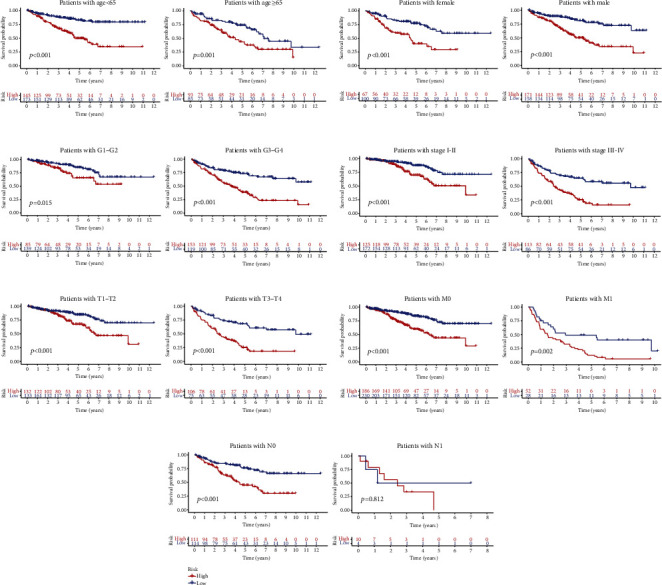
The Kaplan-Meier curve based on KIRC-ICGscore in subsets of different clinical characteristics. (a) Age. (b) Gender. (c) Grade. (d) Stage. (e) T. (f) M. (g) N.

We performed the Kaplan-Meier curves for OS in subsets of different clinical characteristics such as age, gender, stage, grade, and TNM stage (Figures 6(a)–6(g), respectively). We observed the ICG-high subgroup was consistently relevant to adverse prognosis in different subsets except for N1, which might mean that the KIRC-ICG model could independently predict the survival outcome of KIRC patients regardless of clinical features.

### 6.4. Analysis of Tumor Immune Microenvironment (TIME) in KIRC-ICG Subgroups

**Figure 7:**
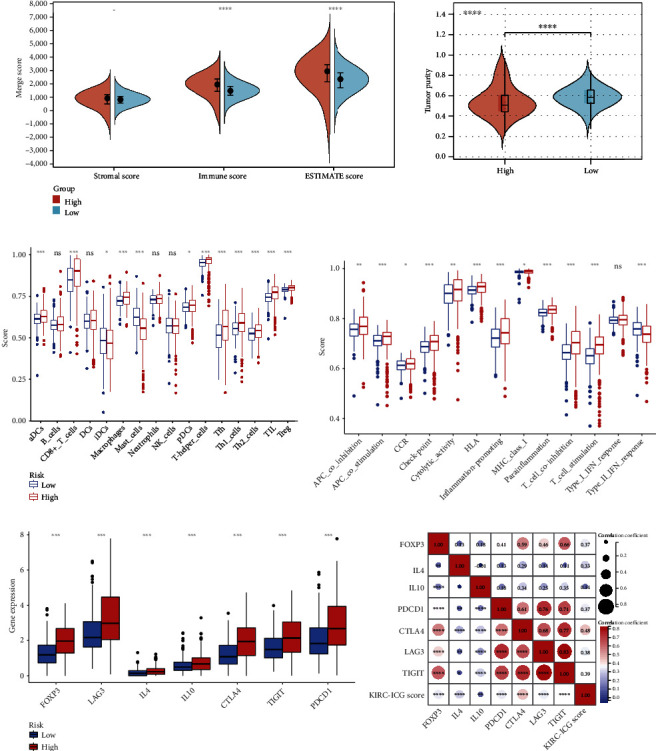
Analysis of TIME in KIRC-ICG subgroups. (a) Differences in the stromal score, immune score, ESTIMATE score, and tumor purity between the two KIRC-ICG subgroups. Differences in immune cells (b) and immune activity (c) between the two KIRC-ICG subgroups. (d) Differential expression of Treg-related markers in the two KIRC-ICG subgroups. (e) Correlation analysis between Treg-related markers and KIRC-ICGscore. ^∗^*p* < 0.05, ^∗∗^*p* < 0.01, ^∗∗∗^*p* < 0.001, and ^∗∗∗∗^*p* < 0.0001.

By applying the “GSVA” and “Estimate” packages, the ICG-high subgroup was revealed to have higher immune and ESTIMATE scores but lower tumor purity status (*p* < 0.05, Figure 7(a)). The ssGSEA analysis demonstrated that the infiltration degrees of dendritic cells (aDCs and pDCs), CD8+ T cells, macrophages, T helper cells (Th1 cells and Th2 cells), follicular helper T cells (Tfh), and regulatory T cells (Treg) were higher in ICG-high subgroup than in the ICG-low subgroup (*p* < 0.05) (Figure 7(b)). Compared to the ICG-low subgroup, the ICG-high subgroup had more immune activities of antigen-presenting cell (APC) coinhibition, APC costimulation, chemokine receptor (CCR), checkpoint, cytolytic activity, human leukocyte antigen (HLA), inflammation-promoting, parainflammation, T cell coinhibition, T cell costimulation, and type II IFN response (Figure 7(c)).

Regulatory T cells (Tregs) are a group of lymphocytes that negatively regulate the immune response and are associated with tumor cells evading immune surveillance. To check the status of Tregs, we compared the expression of the FOXP3 (a marker for Tregs), IL-4, IL-10 (cytokines secreted by Tregs), LAG3, CTLA4, TIGIT, and PDCD1 (coinhibitory molecules expressed by Tregs) between the two KIRC-ICG subgroups. The result showed that their expression was significantly upregulated in the ICG-high subgroup (*p* < 0.05, Figure 7(d)). We further explored the correlation between the KIRC-ICGscore and markers of Tregs and plotted the spearman correlation matrix. The results suggested that the KIRC-ICGscore was significantly positively correlated with the expression of Treg markers of FOXP3, IL-4, LAG3, CTLA4, TIGIT, and PDCD1, except for IL-10 (Figure 7(e)).

### 6.5. The Impact of the KIRC-ICG Model on Immune Therapy Response

**Figure 8:**
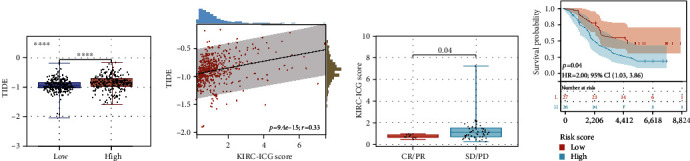
The impact of the KIRC-ICG model on immune therapy response. (a) Difference in TIDE score between the two KIRC-ICG subgroups. (b) Correlation between TIDE score and KIRC-ICGsore. (c) Difference in KIRC-ICGscore between CR/PR and SD/PD group in the IMvigor210 cohort. (d) The Kaplan-Meier curve between the two KIRC-ICG subgroups after atezolizumab therapy. ^∗∗∗∗^*p* < 0.0001.

To check the impact of the KIRC-ICG model on immune therapy, we applied the TIDE algorithm. With correlating gene expression profiles of cytotoxic T lymphocyte (CTL) markers (CD8A, CD8B, GZMA, GZMB, and PRF1) with T-cell characteristics, the TIDE algorithm predicts immunotherapy of tumor patients. The high TIDE score implies that the patient is not responding well to immune therapy due to T-cell dysfunction or more exclusion of T-cell infiltration. The results revealed that the ICG-high subgroup was characterized by significantly higher TIDE scores (Figure 8(a)), although the KIRC-ICGscore had a weak correlation with the TIDE score (*r* = 0.33, *p* < 0.01, Figure 8(b)), which mean that the ICG-high subgroup would have a worse response on immune therapy. By analyzing IMvigor210 data of 64 KIRC patients which were classified as progressive disease (PD), stable disease (SD), partial response (PR), and complete response (CR) according to the treatment response of atezolizumab (PD-L1 inhibitor), we got the result that the CR/PR group had significantly lower KIRC-ICGscore than the SD/PD group (Figure 8(c)). The Kaplan-Meier curve confirmed that the ICG-high subgroup was correlated with worse OS after atezolizumab treatment (Figure 8(d)).

## 7. Construction and Validation of Immue Clustering of KIRC

**Figure 9:**
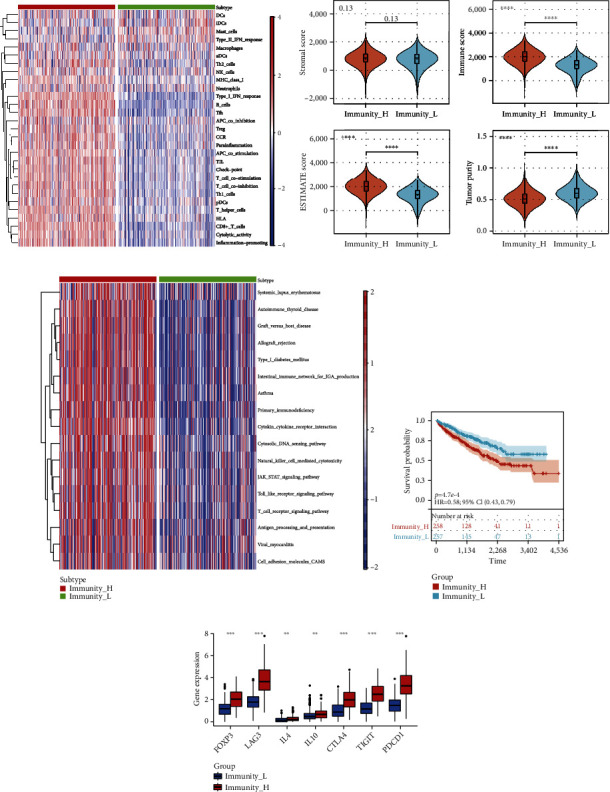
Construction and validation of immune clustering of KIRC. (a) The enrichment levels of 29 immune-related cells and types in the Immunity_H and Immunity_L cluster. (b) Differences in the stromal score, immune score, ESTIMATE score, and tumor purity between the two clusters. (c) GSVA enrichment analysis revealed the different activated biological pathways. (d) The Kaplan-Meier curve between the two clusters. (e) Differential expression of Treg-related markers in the two clusters. ^∗^*p* < 0.05, ^∗∗^*p* < 0.01, ^∗∗∗^*p* < 0.001, and ^∗∗∗∗^*p* < 0.0001.

Based on the ssGSEA algorithm, KIRC patients were analyzed using 29 immune gene sets. The hierarchical clustering algorithm classified KIRC patients (*n* = 515) into the Immunity_H (*n* = 258) and Immunity_L clusters (*n* = 257, Figure 9(a)) based on ssGSEA results. The ESTIMATE algorithm was used to score the TIME of each patient. The results showed that Immunity_H cluster had higher immune and ESTIMATE scores but lower tumor purity status (*p* < 0.05, Figure 9(b)). Then, GSVA enrichment analysis revealed that immune­related pathways such as T-cell receptor signaling pathway, cytokine-cytokine receptor interaction, and primary immunodeficiency were significantly enriched in the Immunity_H cluster (Figure 9(c)). The above results indicated that the Immunity_H cluster had higher immune activities and more immune cell infiltration. The Kaplan-Meier analysis demonstrated that patients in the Immune_H cluster had a poorer prognosis than Immune_L cluster (Figure 9(d)). Meanwhile, cytokines (IL-4 and IL-10) and checkpoints (LAG3, CTLA4, TIGIT, and PDCD1) related to immune suppression were highly expressed in the Immunity_H cluster. The FOXP3 expression was also upregulated in the Immunity_H cluster, which may be attributed to the infiltration of Tregs (Figure 9(e)).

## 8. Discussion

Conventional radiation therapy and cytotoxic chemotherapy have little efficacy in advanced KIRC [5]. Immunotherapy is proven to be a big progression, such as PD-1/PD-L1 inhibitors. PD-1/PD-L1 is one of the immune checkpoint genes that could impair the killing ability of T cells [20]. However, not all patients with PD-L1 overexpression can benefit from PD-L1 inhibitors [21, 22]. Novel ICGs have been discovered [23]. In our study, we collected 47 ICGs from published literature.

Inside the 47 ICGs, cytotoxic T lymphocyte-associated antigen 4 (CTLA-4) belongs to the same immunoglobulin supergene family as CD28. It is mainly expressed on activated T cells and regulatory T cells. With ligands of B7-1 and B7-2 on APCs, CTLA-4 is one of the most important T cell costimulatory receptors [24], and it is considered as a negative regulator of T cells [25]. High expression of CTLA4 was reported to associate with poor overall survival (OS) and might serve as prognostic biomarkers in KIRC patients [26].

Tumor necrosis factor superfamily member 14 (TNFSF14) is mainly expressed on activated T cells, NK cells, and immature dendritic cells (DCs) [27], and its two primary functional receptors are Herpes Virus Entry Mediator (HVEM) and Lymphotoxin-*β* Receptor (LT*β*R). LT*β*R mainly expressed on the surface of epithelial, immature DC, stromal, and myeloid cells [28]. TNFSF14-LT*β*R interaction is necessary for lymphoid structure development and maintenance [29]. However, it is expressed on lymphocytes, NK cells, and smooth muscle; HVEM functions is an important T-cell costimulatory factor [30, 31]. TNFSF14-HVEM can induce strong antitumor immunity in a therapeutic context [32]. TNFSF14 was reported to be a prognostic factor as one of the TNF-related signatures and could assess the efficacy of immunotherapy [33].

Human endogenous retrovirus-H long terminal repeat-associating protein 2 (HHLA2) is a new member of the B7 family and could mediate costimulation by interacting with TMIGD2 [34]. Patients with HHLA2 overexpression exhibited a worse clinical prognosis in KIRC [35]. HHLA2 overexpression is also found in other carcinomas like intrahepatic cholangiocarcinoma [36], stomach cancer [37], and colorectal cancer [38] and is associated with poor prognosis. However, there were also reports that HHLA2 was highly expressed in KIRC and predicted a favorable survival outcome in KIRC [39, 40], which was consistent with our research. According to the report, HHLA2 has a costimulatory receptor TMIGD2 (transmembrane and immunoglobulin domain containing 2) and a coinhibitory receptor KIR3DL3 (killer cell Ig-like receptor, three Ig domains, and long cytoplasmic tail), which endows it with both immunostimulant and immunosuppression functions in cancer development [41]. These opposing functions emphasized that the expression and distribution of HHLA2 and its receptor may determine the immune response in the tumor microenvironment, thereby influencing prognosis.

Together with three ICGs (CTLA4, TNFSF14, and HHLA2), here, we successfully established the predicting KIRC-ICG model for the first time. Based on the IMvigor210 cohort, we proved that patients in the ICG-high subgroup were less sensitive to ICGs. Meanwhile, we applied the TIDE algorithm to speculate the reason why the ICG-high subgroup was less sensitive to ICIs from the perspective of the CTL. The result showed the TIDE score in the ICG-high subgroup that was higher than the ICG-low subgroup, which indicated that the patients are not responding well to immune therapy due to more T-cell dysfunction or more exclusion of T-cell infiltration. Notably, the KIRC-ICGscore only weakly correlated with TIDE scores. We hypothesized that in addition to the CTL, there may be other immune cells that influence the immunotherapy response by interacting with CTL. Even though there are limitations on the bioinformatic model for prediction prognosis and immunotherapy response, it could give us a different perspective on the function of the corresponding risk factors.

Contrary to the general trend of tumor immunity, patients with poorer prognosis had higher immune activities and more immune cell infiltration (including Tregs) in our study. To further verify this opposite immune characteristic, the ssGSEA algorithm was applied. By the ssGSEA algorithm, KIRC patients were classified into the Immunity_H and Immunity_L clusters. And the Immune_H cluster had shorter OS and more Treg cell infiltration than those in the Immune_L cluster, which was the same as the immune characteristic of the KIRC-ICG model. Some reports have proved that high tumoral immune activity does not necessarily indicate a better prognosis in certain cancer types [42, 43]. The above trend in cancer immunology is uncommon, and it may be influenced by cancer-type-specific factors. According to the report, high immune activity in uveal melanoma might induce epithelial-mesenchymal transition/endothelial-mesenchymal transition and loosen the blood barrier structure, further leading to a poor prognosis [44]. However, the underlying mechanisms of the special immune characteristic of KIRC are unclear and needed to be further explored.

## 9. Conclusion

In conclusion, we created a new KIRC-ICG model which was reliable to predict prognosis and immunotherapy response for KIRC patients and could be an independent factor regardless of clinical characteristics. However, this study still has some limitations. To further validate the model, bench experiments are needed in the future.

## Figures and Tables

**Table 1:** Details of 47 immune checkpoint genes.

47 Immune checkpoint genes
IDO1, LAG3, CTLA4, TNFRSF9, ICOS, CD80, PDCD1LG2, TIGIT, CD70, TNFSF9, ICOSLG, KIR3DL1, CD86, PDCD1, LAIR1, TNFRSF8, TNFSF15, TNFRSF14, IDO2, CD276, CD40, TNFRSF4, TNFSF14, HHLA2, CD244, CD274, HAVCR2, CD27, BTLA, LGALS9, TMIGD2, CD28, CD48, TNFRSF25, CD40LG, ADORA2A, VTCN1, CD160, CD44, TNFSF18, TNFRSF18, BTNL2, CD200R1, TNFSF4, CD200, NRP1, C10orf54

## Data Availability

All data mentioned in this paper can be downloaded from TCGA (https://portal.gdc.cancer.gov) and ICGC (http://dcc.icgc.org/) databases and IMvigor210 dataset (http: http://research-pub.gene.com/IMvigor210CoreBiologies).
